# Dual Evidence of *Trichoderma pleuroti* Antagonism against *Pleurotus pulmonarius*: Direct Inhibition and Volatile-Mediated Bioassays with Volatilome Fingerprinting

**DOI:** 10.4014/jmb.2605.05010

**Published:** 2026-09-21

**Authors:** Wan Swan Shan, Kah Hin Low, Yingying Wu, Yee Shin Tan

**Affiliations:** 1 Mushroom Research Centre, Universiti Malaya, 50603 Kuala Lumpur, Malaysia; 2 Institute of Biological Sciences, Faculty of Science, Universiti Malaya, 50603 Kuala Lumpur, Malaysia; 3 Department of Chemistry, Faculty of Science, Universiti Malaya, 50603 Kuala Lumpur, Malaysia; 4 National Engineering Research Center of Edible Fungi, Key Laboratory of Applied Mycological Resources and Utilization of Ministry of Agriculture, Institute of Edible Fungi, Shanghai Academy of Agricultural Sciences, Shanghai, P. R. China

**Keywords:** Green mold contamination, Inhibition, Mushroom, Mycoparasitism, Volatile Organic Compounds (VOCs)

## Abstract

*Trichoderma* is known to cause green mold contamination, which severely threatens *Pleurotus pulmonarius* cultivation in tropical regions, including Malaysia, and can lead to major yield and economic losses. In this study, the contact-dependent and contactless antagonistic interactions between *Trichoderma pleuroti* and *P. pulmonarius* were investigated, and associated morphological changes and volatile profiles were characterized. Dual-culture assays recorded rapid overgrowth of *P. pulmonarius* by *T. pleuroti*, leading to growth suppression and mycelial discoloration. Light and scanning electron microscopy demonstrated aggressive mycoparasitism in *T. pleuroti*, which showed hyphal deformation and specialized structure attachment at the interaction interface. Inverted and tripartite plate methods were used to evaluate volatile-mediated effects. In *P. pulmonarius*, exposure to *T. pleuroti* volatiles induced abnormal mycelial thickening and growth inhibition, consistent with a contribution of fungal volatiles to contactless antagonism. Headspace gas chromatography-ion mobility spectrometry revealed distinct volatilome patterns and identified several candidate VOC features associated with *T. pleuroti*, including 1-octanol, 1-octen-3-ol, 3-octanol, (E)-2-hexenal, (E)-2-heptenal, (E)-2-octenal, acetic acid and butanoic acids. Overall, *T. pleuroti* exhibited both contact-dependent and contactless antagonistic effects against *P. pulmonarius* mycelium, involving mycoparasitic interactions and volatile-mediated inhibition. These findings provide insights into the development of green mold disease in *Pleurotus* cultivation and contribute to a better understanding of fungus-fungus interactions in mushroom production.

## Introduction

*Pleurotus* species, commonly known as oyster mushrooms, are among the most widely cultivated edible fungi due to their nutritional value, low sugar and fat content, and abundance of health-promoting bioactive compounds [1]. In Malaysia, *Pleurotus pulmonarius* (grey oyster mushroom) is the most popular cultivated species and contributes substantially to local production [2]. At the national level, mushroom production remains economically relevant, with recent statistics reporting a total output of 8,445.3 metric tons in 2024 across Malaysia [3]. This production landscape illustrates the value of supportive management and biosecurity practices for sustaining cultivation. In parallel, mushrooms, including oyster mushrooms, have been positioned within Malaysia’s high-value agrofood agenda [4] and remain aligned with the National Agrofood Policy 2021–2030 (NAP 2.0) [5], which emphasizes sustainability, resilience, and technology-driven growth along with food security and nutrition.

However, the sustainability of *Pleurotus* cultivation is frequently threatened by green mold diseases, most commonly associated with *Trichoderma* species that compete aggressively for nutrients and space and can cause substantial yield losses; for example, up to 78.6% of *P. eryngii* production has been reported [6]. Globally, these outbreaks have caused severe economic consequences, including instances of total production failure [7]. In Malaysia, *T. pleuroti*, *T. harzianum*, and *T. ghanense* have been isolated from contaminated cultivation environments of *P. pulmonarius*, with *T. pleuroti* identified as the predominant agent in affected farms [8]. Earlier findings also supported that *T. pleuroti* constitutes ~94% of *Trichoderma* isolates recovered from contaminated sawdust substrates used for *Pleurotus* cultivation [9]. The temporal occurrence of these species appears at different times; *T. pleuroti* is often detected at later cultivation stages (*e.g.*, days 20–30 and postharvest) from infected bags, water sources, and the farm environment, whereas *T. harzianum* and *T. ghanense* are reported more frequently during the early stages. Consistent with its epidemiological importance, *T. pleuroti* exhibits a strong colonization capacity and can rapidly overgrow *Pleurotus* mycelia, unlike other *Trichoderma* spp. that remain localized [8, 10].

*Trichoderma* antagonism can involve both direct and indirect mechanisms. Direct mycoparasitism includes host recognition, chemotrophic growth, hyphal contact, and the secretion of lytic enzymes and secondary metabolites that degrade host cell walls disrupt cellular integrity, sometimes aided by specialized structures like appressoria [11, 12]. In addition to contact-dependent antagonism, *Trichoderma* emits volatile organic compounds (VOCs), a type of diffusible metabolite spanning a variety of chemical classes (*e.g.*, short-chain alcohols, aromatics, and terpenes), that function in signaling and may exert antifungal activity [13]. Volatile-mediated suppression of fungal growth or spore germination has been reported in multiple pathosystems, including effects linked to C_4_-C_8_ alcohols and other *Trichoderma*-associated volatiles [14, 15]. However, volatilome profiles of green-mold-associated *Trichoderma* spp., particularly in the context of *P. pulmonarius* cultivation, remain inadequately characterized.

Therefore, this study aims to (i) evaluate the inhibitory effects of green mold-associated *Trichoderma* isolates on *P. pulmonarius* growth; (ii) characterize dual-culture confrontation outcomes and associated macroscopic/microscopic features to capture contact-dependent antagonism; (iii) assess contactless volatile-mediated effects on *P. pulmonarius* using inverted-plate and tripartite bioassays; and (iv) profile and compare monoculture headspace volatilomes using headspace gas chromatography-ion mobility spectrometry (HS-GC-IMS) to identify candidate discriminatory VOC markers associated with predominant *Trichoderma* isolates.

## Materials and Methods

### Fungal Strains and Culture Conditions

The *Pleurotus pulmonarius* strain, PL27 (KLU-M1392, sourced from a local mushroom farm), and the mycopathogenic strain *Trichoderma pleuroti* D20-21 (Accession no: OR028919) were obtained from a previous study [8]. *Trichoderma asperellum* A11 (Accession no: OR028879), a non-dominant *Trichoderma* species frequently found in mushroom substrate, was utilized as a reference strain for volatilome profiling [8]. All fungal strains were maintained on potato dextrose agar (PDA; OXOID) and incubated at 28 ± 2°C. For this study, 3-day-old *Trichoderma* cultures and 7-day-old *P. pulmonarius* cultures were used for subsequent assays to examine differences in growth kinetics and VOC emission timing.

### *In Vitro* Direct Plate Assay

The direct inhibitory effect of *Trichoderma* on *P. pulmonarius* was evaluated using a dual-culture confrontation method [16]. A culture plug of *P. pulmonarius*, 10 mm in diameter, was inoculated on one side of a PDA plate and incubated for 3 days. A 10 mm *Trichoderma* culture plug was then placed on the opposite side of the same plate (Fig. 1A). Plates were incubated at 28 ± 2°C for 7 days. The radial mycelium growth of both fungi was measured using a ruler, and visible pigmentation or discoloration and interaction features at the confrontation zone were recorded. Individual monocultures of *P. pulmonarius* and *Trichoderma* species grown on separate plates under identical incubation conditions served as controls. The experiment was conducted in biological triplicates.

### Individual Inhibitory Effect (IIE), General Inhibitory Effect (GIE), and Inhibitory Rating

Dual-culture interaction outcomes were evaluated using the individual inhibitory effect (IIE) approach [17], which considers colony encirclement/overgrowth, inhibition zones, and reduced colony development (Table S1). A general inhibitory effect (GIE) value was subsequently derived by combining the IIE score with the frequency of each occurrence. Additionally, an inhibitory rating was assessed using a scale [18], with modifications (Table S1). The inhibitory index (II) was calculated as:

II = A n × 1()Bn×2()++B_A1_ ×n2.()5 B_A2_+×n3().5

In this context, n represents the frequency of observations for each reaction type or subtype, while A, B, BA1, and BA2 denote the inhibitory reaction types recorded in the direct plate assay found in Table S1. The relative frequency (ƒ, %) of each inhibition type/subtype was calculated as:

$$\text{f=}\frac{\text{score of a given reaction type/subtype}}{\text{total inhibitory index}}\times 100$$

### *In Vitro* Inverted Plate Assay

The effects of volatile compounds released by *Trichoderma* spp. on the growth of *P. pulmonarius* were evaluated using an inverted plate assay [19] with modifications. One PDA plate was inoculated with a 10 mm *Trichoderma* mycelial plug, while another PDA plate inoculated with a 10 mm *P. pulmonarius* mycelial plug was inverted and placed over the *Trichoderma*-inoculated plate (Fig. 1B). Both plates were used without lids, sealed together with parafilm to minimize VOC loss, and incubated at 28 ± 2°C for 7 days. Control treatments included *P. pulmonarius* or *Trichoderma* strains cultured alone under identical conditions. The experiment was performed in biological triplicate.

### *In Vitro* Tripartite Plate Assay

The tripartite plate assay was conducted using a three-compartment Petri dish (Nest Biotechnology) (Fig. 1C) [20]. A 3-compartment Petri dish was used to inoculate the following components: a sterilized sawdust substrate, a 10 mm diameter *Trichoderma* plug (pathogenic or non-pathogenic) on PDA, and a 10 mm diameter *Pleurotus* plug cultured on PDA. The role of the sterilized sawdust substrate was to mimic commercial cultivation conditions and allow VOC interaction in a more compartmentalized setup. Plates were incubated at 28 ± 2°C for 7 days. Mycelial growth was measured at 24-h intervals until the relevant control condition reached full colonization. Control setups included (i) PDA cultures of *Trichoderma* target strain alone, (ii) PDA cultures of *Trichoderma* reference strain alone, (iii) PDA cultures of *P. pulmonarius* alone, (iv) sterilized sawdust alone, and (v) a combination of *P. pulmonarius* with sterilized sawdust. The experiment was conducted in triplicate.

### Percentage Growth Inhibition

The growth rate of each culture was measured, and the percentage inhibition of mycelial growth of *P. pulmonarius* in the direct and inverted plate assay was calculated [21]:

$$\text{Percentage inhibition (\%)}=\frac{D_{1}-D_{2}}{D_{1}}\times 100$$

where *D*_1_ is the radial mycelial growth of *P. pulmonarius* in monoculture (control), and D2 is the mycelial growth of *P. pulmonarius* when co-cultured with *Trichoderma*.

### Light Microscopy and Scanning Electron Microscopy (SEM)

Microscopic analysis followed [22]. Mycelia from the interaction zone of the confrontation assay were sampled on the 7^th^ day of incubation, stained with methylene blue, mounted on a microscope slide and examined under an Olympus microscope at 400 × magnification to observe early interaction features. In scanning electron microscopy analysis, a 9 mm^2^ plug from the interaction zone in the direct plate assay on the 3rd day post-inoculation was excised and processed for fixation, dehydration, and coating. Samples were fixed using osmium oxide and sequentially dehydrated through ethanol followed by acetone. Processed samples were performed in triplicate and were dried and sputter-coated with gold (Cressington 108) before SEM examination (PHENOM proX).

### Volatile Organic Compounds Profiling by HS-GC-IMS

VOCs were profiled by HS-GC-IMS following [23] with minor modifications. Approximately 1.0 g of mycelium from each monoculture (*P. pulmonarius*, *T. pleuroti*, and *T. asperellum*) previously grown on PDA were transferred into 20-mL headspace vials; uninoculated PDA served as the blank control. 2-methyl-3-heptanone (10 μL of 100 μg/mL) was added as an internal standard (IS), and vials were incubated at 45°C for 40 min prior to injection. Using an autosampler (CTC Analytics AG, Switzerland), 500 μL headspace was injected at 85°C into an Agilent 490 GC coupled with a FlavourSpec^®^ IMS. Chromatographic separation was performed on a MXT-WAX column (30 m × 0.53 mm × 1.0 μm; Restek, USA) at 60°C with N_2_ carrier gas programmed as follows: 2.0 mL/min (0−2 min), increase linearly to 10.0 mL/min (2-10 min), then ramp to 100 mL/min over 10 min and hold until 55 min. The IMS drift tube operated at 45°C with N_2_ drift gas at 75 mL/min. All measurements were conducted in biological triplicate.

Retention indices (RI) were calculated using an n-ketone series (2-butanone to 2-nonanone). Peaks were putatively identified by matching RI and drift time against the NIST/GC-IMS library [24]. Semi-quantitative concentrations were estimated by internal-standard scaling:

$${\text{C}}_{\text{t}}\left({\text{mg kg}}^{\text{-1}}\right)\text{=}\frac{{\text{C}}_{\text{i}}\left({\text{mg μL}}^{\text{-1}}\right){\text{×V}}_{\text{i}}\left(\text{μL}\right){\text{×PV}}_{\text{t}}}{{\text{PV}}_{\text{i}}{\text{×m}}_{\text{t}}\left(\text{kg}\right)}$$

In this context, Ct represents the estimated concentration of the target compound, while *C_i_* and *V_i_* refer to the concentration and volume of the internal standard (IS); *PV_t_* and *PV_i_* denote the peak volumes of the target compound and the IS, respectively; and *m_t_* indicates the sample mass. Raw data were processed in Laboratory Analytical Viewer (LAV) software (version 2.2.1).

### Statistical Analysis

Univariate analyzes were performed in IBM^®^ SPSS^®^ Statistics 26. Experimental data were analyzed by one-way analysis of variance (ANOVA), and Duncan's multiple range test (DMRT) was applied for post-hoc comparisons (least significant difference-based) at the 95% confidence level.

Multivariate analyzes were conducted in SAS^®^ JMP^®^ Statistical Discovery (version 18.2.1). Principal component analysis (PCA) was computed on the correlation matrix for unsupervised exploration. Hierarchical cluster analysis (HCA) uses Euclidean distance and Ward’s linkage for standardized variables. Partial least squares-discriminant analysis (PLS-DA) was performed on autoscaled data using the nonlinear iterative partial least squares (NIPAL) algorithm, with the number of latent variables (LVs) optimized using leave-one-out cross-validation. Model performance was evaluated using Q^2^, R^2^X, and R^2^Y. To assess the statistical significance of the observed class separation and the potential overfitting, permutation testing was performed using 1000 permutations of the class label. Variable importance in projection (VIP) scores greater than 0.8, together with model coefficients were used to identify variables contributing to class separation.

## Results and Discussion

### Direct Antagonism of *P. pulmonarius* by *Trichoderma*

In this study, the dominant outbreak-associated *T. pleuroti* was evaluated for its aggressive antagonism toward *P. pulmonarius* [8], while *T. asperellum* served as a reference strain based on prior reports describing a deadlock-type interaction upon mycelial contact and comparatively mild morphological effects on *Pleurotus* mycelium [25]. This comparative design establishes an in vitro standard and aligns with findings that *T. pleuroti* is the primary green mold agent in the cultivation of *P. pulmonarius* in Malaysia [9].

Macroscopically, dual-culture confrontation assays showed that *Trichoderma* spp. markedly affected *P. pulmonarius* growth following colony contact (Fig. 2). *T. pleuroti* rapidly advanced towards *P. pulmonarius*, causing early growth suppression and visible discoloration at the interaction front, and subsequently overgrew the host colony within 3 days (Fig. 2B). This rapid colonization is consistent with reports that *T. pleuroti* exhibits a strong competitive ability and can outcompete mushroom mycelia for space and nutrients during green mold outbreaks [8]. The yellowing symptom was first observed at the contact zone and later intensified to brown pigmentation, which may reflect stress-associated physiological responses reported in fungi during antagonistic interactions, including altered secondary metabolism and extracellular pigment production [26]. In contrast, when co-cultured with *T. asperellum*, *P. pulmonarius* exhibited relatively mild symptoms, such as localized browning and mycelial thickening, without complete host replacement after 7 days (Fig. 2C), indicating a weaker inhibitory effect compared with *T. pleuroti*.

Microscopically, clear differences in interaction were observed between the two *Trichoderma* species. When *T. pleuroti* was present, the *Pleurotus* hyphae at the interaction zone recorded thin, short, and sparsely branched, and clamp connections were rare (Fig. 2B). This suggests that normal dikaryotic hyphal development was disrupted. Such disruptions are consistent with reports that severe green mold infections can compromise mycelial development and fruiting [27]. Hyphal damage was also evident at the interaction region, consistent with lysis/enzyme-assisted mycoparasitism reported for *Trichoderma* spp. [11]. Scanning electron microscopy further supported aggressive contact-dependent antagonism by *T. pleuroti*, where hyphal swelling and specialized structure attachment were observed at the interface (Fig. 2B), facilitating adhesion and penetration of host hyphae during mycoparasitic attack [28]. However, the interaction with *T. asperellum* was mainly characterized by hyphal coiling with limited structure damage (Fig. 2C), consistent with a deadlock-type interaction rather than rapid host replacement. Similar coiling and localized host responses have been reported in less aggressive antagonistic encounters and may reflect a stress- or defense-associated morphological response to the host mycelia [29].

Quantitatively, inhibition metrics supported the stronger antagonism of *T. pleuroti* compared with *T. asperellum*. According to the interaction scoring and inhibitory index classification, *T. pleuroti* most frequently exhibited mycelial replacement (II: type BA2) and complete suppression of *P. pulmonarius* development (IIE: +4), whereas *T. asperellum* more commonly resulted in a deadlock interaction (IIE: 0 and II: type A) (Table 1). Consistent with these interaction outcomes, *T. pleuroti* caused the highest growth inhibition of *P. pulmonarius* (~97%), while *T. asperellum* produced lower inhibition (≤62%) (Fig. 3). Overall, macroscopic, microscopic, and quantitative evidence shows that *T. pleuroti* has stronger contact-dependent inhibition against *P. pulmonarius* than the non-dominant comparator strain under the tested conditions, which is consistent with its reported epidemiological relevance in green mold outbreaks.

### Indirect Inhibition of *P. pulmonarius* by *Trichoderma*

Since *Trichoderma* antagonism involves both contact-dependent mycoparasitism and volatile/airborne effects [12], and contactless impacts on

*P. pulmonarius* growth were assessed using inverted and tripartite bioassays (Fig. 4). In the inverted plate assay, *P. pulmonarius* exposed to headspace volatiles from *T. pleuroti* showed a significant growth inhibition of 14% (Fig. 3), along with growth delay and abnormal mycelial thickening, even without physical contact (Fig. 4B). This phenotype is consistent with the volatile-mediated antagonism reported in fungus-fungus interactions, in which VOCs may potentially act as airborne modulators of fungal development [12]. Similar inhibitory effects have been seen in other systems; for example, alcohol-type VOCs such as 3-methyl-1-butanol and 2-methyl-1-propanol from T. atroviride have been linked to cell deformation and growth suppression in target fungi [14]. In contrast, exposure to *T. asperellum* had minimal effects on *P. pulmonarius* (0% inhibition) (Fig. 3), with hyphal morphology similar to the control and clamp connections still visible under the tested conditions (Fig. 4C). This comparatively weak contactless effect may reflect lower emission of inhibitory volatiles under the present conditions and is consistent with reports that inhibition by some *Trichoderma* strains can be more pronounced when contact-dependent mechanisms are available [30].

In the tripartite plate assay, contactless interactions were evaluated in the presence of sterilized sawdust, a commonly used cultivation substrate for *P. pulmonarius*, to mimic farm-relevant conditions (Fig. 4D-4F). In this assay, *P. pulmonarius* likewise exhibited growth suppression in the presence of *T. pleuroti*, which became apparent after day 6 (Fig. 4E), despite physical separation between fungal compartments. This observation supports the involvement of airborne/diffusible factors under contactless conditions. Notably, *T. pleuroti* also vigorously colonized the sawdust compartment during incubation, which is consistent with their strong substrate competitiveness under resource-limited conditions and aligns with reports that *Trichoderma* spp. can influence neighboring fungal growth via diffusible signals [8, 31]. In contrast, *T. asperellum* produced no measurable suppression; *P. pulmonarius* mycelium extended and colonized the sawdust substrate, whereas *T. asperellum* showed only the minimum substrate colonization (Fig. 4F).

### Volatilome Fingerprinting and Discriminatory VOC

Headspace VOCs from monocultures of *P. pulmonarius* (PL27), *T. pleuroti* (D20-21), and *T. asperellum* (A11) grown on PDA were profiled using HS-GC-IMS to compare the host volatilome with that of an outbreak-associated antagonist and a non-dominant comparator, consistent with the antagonism phenotypes observed in plate assays (Fig. 5). The 2D and 3D topography (Fig. 5A and 5B), with *Trichoderma*, particularly *T. pleuroti*, showing higher and more extensive signal intensities than *P. pulmonarius* under the conditions tested. This pattern suggests greater overall volatilome output by *T. pleuroti* during PDA growth and is directionally consistent with its stronger antagonism phenotype observed in confrontation and contactless bioassays.

To interpret the chemical basis underlying these pattern-level differences, detected signals were putatively annotated using RI and drift-time matching. Across samples, 68 VOCs were identified, and 10 signals remained unidentified. Identified VOCs comprised 14 alcohols, 18 aldehydes, 1 amine, 3 carboxylic acids, 12 esters, 4 furans, 10 ketones, 1 other nitrogen-containing compound, 3 sulphur-containing compounds, and 2 terpenes (Fig. S1; Table S2). Alcohols, aldehydes, and ketones were the dominant classes across both *P. pulmonarius* and *Trichoderma*, consistent with typical fungal volatilomes in which these compounds arise from central carbon metabolism and lipid-derived pathways and are readily detected in headspace analyzes [32].

A gallery fingerprint plot provided a feature-level overview of VOC patterns across PDA controls and fungal monocultures (Fig. 5C). PDA blanks exhibited uniformly low intensities, indicating limited medium-derived background under the acquisition conditions. In contrast, fungal samples displayed broader and stronger feature patterns, and *P. pulmonarius* profiles were visually distinct from the two *Trichoderma* species, whereas the two *Trichoderma* isolates showed greater overall similarity, supporting downstream multivariate analyses to formalize group structures and identify discriminatory features.

PCA provided an unsupervised summary of VOC variation across groups (Fig. 6A). Along PC1 (38.9% of total variance), PDA and *P. pulmonarius* lay predominantly on the negative side, whereas both *Trichoderma* isolates projected on the positive side, indicating that the dominant axis of variance reflects a shift from medium/background and host-associated profiles toward *Trichoderma*-associated volatilome output. PC2 (18.7% of total variance) further resolved differences between *T. pleuroti* and *T. asperellum*, while *P. pulmonarius* remained separable from PDA, supporting an active host volatilome above the medium baseline. Consistent with this separation, loading vectors suggested that *T. pleuroti* aligned with features such as short-chain carboxylic acids, unsaturated aldehydes, and selected C8-type volatiles, whereas *T. asperellum* aligned more strongly with several alcohol-type features and related volatiles. *P. pulmonarius* aligned with a different subset (*e.g.*, benzaldehyde and 2-methyl-butenal), supporting host-association VOC production. Overall, the biplot indicates that separation is driven by coordinated abundance shifts across multiple VOC features rather than a single compound [13].

HCA complements PCA by visualizing sample similarity as a dendrogram (Fig. 6B). PDA blanks clustered tightly and remained separated from fungal samples, reinforcing that medium-derived background did not account for the observed patterns. *P. pulmonarius* formed a distinct cluster separated from the *Trichoderma* branch, while *T. pleuroti* and *T. asperellum* clustered closer to each other than to *P. pulmonarius*, indicating higher within-genus similarity in volatilome output. The proximity of the *T. pleuroti* replicates to the *P. pulmonarius* cluster suggests that there is replicate-level variability inherent to headspace profiling; the remaining *T. pleuroti* replicates grouped within the *Trichoderma* branch, supporting an overall stable species-level structure.

To move from pattern-level separation toward interpretable chemical drivers, PLS-DA models were applied to identify VOC features contributing to class discrimination. Cross-validation selected five LVs for the model, with R^2^X of 0.880, R^2^Y of 0.997, and Q^2^ of 0.913, indicating high explained variance and cross-validated predictive performance [33]. Permutation testing with 1000 random permutations yielded a p-value of 0.004, indicating that the observed class separation was unlikely to result solely from random class assignment. Coefficient sign patterns were visualized using a Venn diagram (Fig. 7) to highlight VOCs comparatively associated with PDA backgrounds, *P. pulmonarius*, *T. pleuroti*, or *T. asperellum* samples and to distinguish shared versus class-associated signatures. *P. pulmonarius* produced a comparatively small host-associated set (including benzaldehyde and 4-hexen-3-one), suggesting a candidate *P. pulmonarius*-associated volatilome signature distinct from those of the two *Trichoderma* species. The two *Trichoderma* species showed substantial overlap (*e.g.*, ethanol, 1-butanol, thiazole and several esters/ketones), consistent with their closer similarity observed in PCA and HCA, suggesting these shared outputs may be less informative for differentiating outbreak-associated versus non-dominant isolates under PDA growth conditions.

*Trichoderma pleuroti* was associated with an expanded set of VOC features, including short-chain carboxylic acids, unsaturated aldehydes, C_8_-type volatiles, and specific esters. These compounds are frequently implicated as growth-modulatory or stress-inducing cues in fungus-fungus interactions (Table 2). For example, acetic and butanoic acids have been reported to suppress diverse fungi [14, 34], while (E)-2-hexenal, (E)-2-heptenal, and (E)-2-octenal have been associated with membrane perturbation and oxidative stress-related responses in fungal targets in previous studies [35-37], suggesting possible modes of action that warrant direct validation in future studies. Ester-type volatiles, specifically ethyl acetate and methylbenzoate, have also shown inhibitory activity in microbial systems [38, 39]. Likewise, C_8_-type volatiles (*i.e.*, 1-octen-3-ol, 3-octanol, and 1-octen-3-one) have been reported in previous studies to influence fungal development, including germination inhibition and interspecies chemical communication [15, 40]. The enrichment of these compounds in *T. pleuroti* is directionally consistent with the stronger contactless suppression effects observed in inverted and tripartite assays, identifying them as candidate VOC features that may contribute to airborne inhibition.

The volatile profiling results were compared with existing literature to assess the novelty of the VOCs identified in *T. pleuroti* (Table 1). Several detected compounds have been reported in other *Trichoderma* species, where 1-octanol, 1-octen-3-ol, and ethyl acetate were reported in *T. harzianum* [41]. Compounds such as 3-octanol and methyl benzoate have been detected in T. longibrachiatum [42]. Acetic acid and butanoic acid were reported to be produced by T. virens [41, 42]. Propanoic acid was previously reported for T. afroharzianum [43], while 1-hydroxy-2-propanone was reported in T. koningiopsis [44]. To the best of our knowledge, three of the identified volatiles: (E)-2-heptenal, (E)-2-hexenal, and (E)-2-octenal, have not previously been reported in *T. pleuroti*, suggesting these compounds may represent previously undescribed compounds of its volatilome.

*Trichoderma asperellum* exhibited its own class-associated subset (*e.g.*, α-terpinene and several alcohol/aldehyde), consistent with its separation from *T. pleuroti* samples along PC2. The comparatively smaller *T. asperellum*-specific set, together with weaker suppression under contactless conditions, suggests that differences between the two *Trichoderma* isolates may reflect both quantitative (overall headspace output) and qualitative (VOC composition) shifts rather than a single defining marker. Finally, potato dextrose agar media showed only a limited background set (*e.g.*, furfural, hexanal, and nonanal), supporting that the discriminatory patterns observed in fungal samples were unlikely to be dominated by medium-derived artefacts.

The well-known antifungal lactone 6-pentyl-2H-pyran-2-one (6-PP) was not detected in either *T. pleuroti* or *T. asperellum* under the present conditions. This absence may reflect species- or strain-specificity and the strong dependence on culture conditions, consistent with reports that 6-PP is not universally produced across *Trichoderma* species and can vary with substrate composition, incubation time, and environmental parameters [45]. Although the volatilomes were obtained from monocultures and therefore cannot establish causality, the enrichment of acids, aldehydes, and C_8_-type volatiles in *T. pleuroti* supports their consideration as candidate VOC features for further validation in farm-relevant matrices or controlled exposure experiments.

## Conclusion

This study provides insight into green mold contamination in *P. pulmonarius* cultivation associated with *T. pleuroti* by providing evidence of both contact-dependent and contactless antagonism. In a dual-culture confrontation assay, *T. pleuroti* rapidly colonized the medium, overgrew *P. pulmonarius*, and caused pronounced growth suppression with visible discoloration, whereas *T. asperellum* showed weaker inhibition and typically produced a deadlock-type interaction. Contactless inhibition was supported by inverted and tripartite assays, where *P. pulmonarius* exhibited growth impairment, including abnormal mycelial thickening, in the presence of *T. pleuroti* despite physical separation, and *T. pleuroti* also vigorously colonized the sawdust compartment under cultivation-related conditions. Microscopy further supported aggressive mycoparasitism by *T. pleuroti*, including host hyphal damage and structure attachment at the interface, while reduced clamp connections/generative hyphae in *P. pulmonarius* during confrontation are consistent with disrupted dikaryotic development. HS-GC-IMS profiling of monocultures revealed distinct volatilome signatures and identified candidate VOC features associated with *T. pleuroti*, including 1-octanol, 1-octen-3-ol, 3-octanol, (E)-2-hexenal, (E)-2-heptenal, (E)-2-octenal, acetic acid, and butanoic acid. Several of these VOCs have previously been associated with growth-modulatory effects in fungal interactions, and therefore represent candidate contributors to the contactless effects observed in the volatile-mediated bioassays, although their roles remain to be experimentally validated.

This study provides useful baseline information, although the volatilome analysis was conducted with monoculture samples and may therefore not fully capture interaction-induced VOC dynamics during fungal confrontation. In addition, individual hyphae of *Trichoderma* spp. and *P. pulmonarius* within direct interaction zones were difficult to distinguish because of their morphological overlap. Future work should profile co-culture headspace over time and validate candidate VOCs using pure compounds or controlled exposure assays, supported by targeted microscopy and molecular readouts to establish relationships between VOC exposure to host developmental responses.

## Supplemental Materials

Supplementary data for this paper are available on-line only at http://jmb.or.kr.



## Figures and Tables

**Fig. 1:**
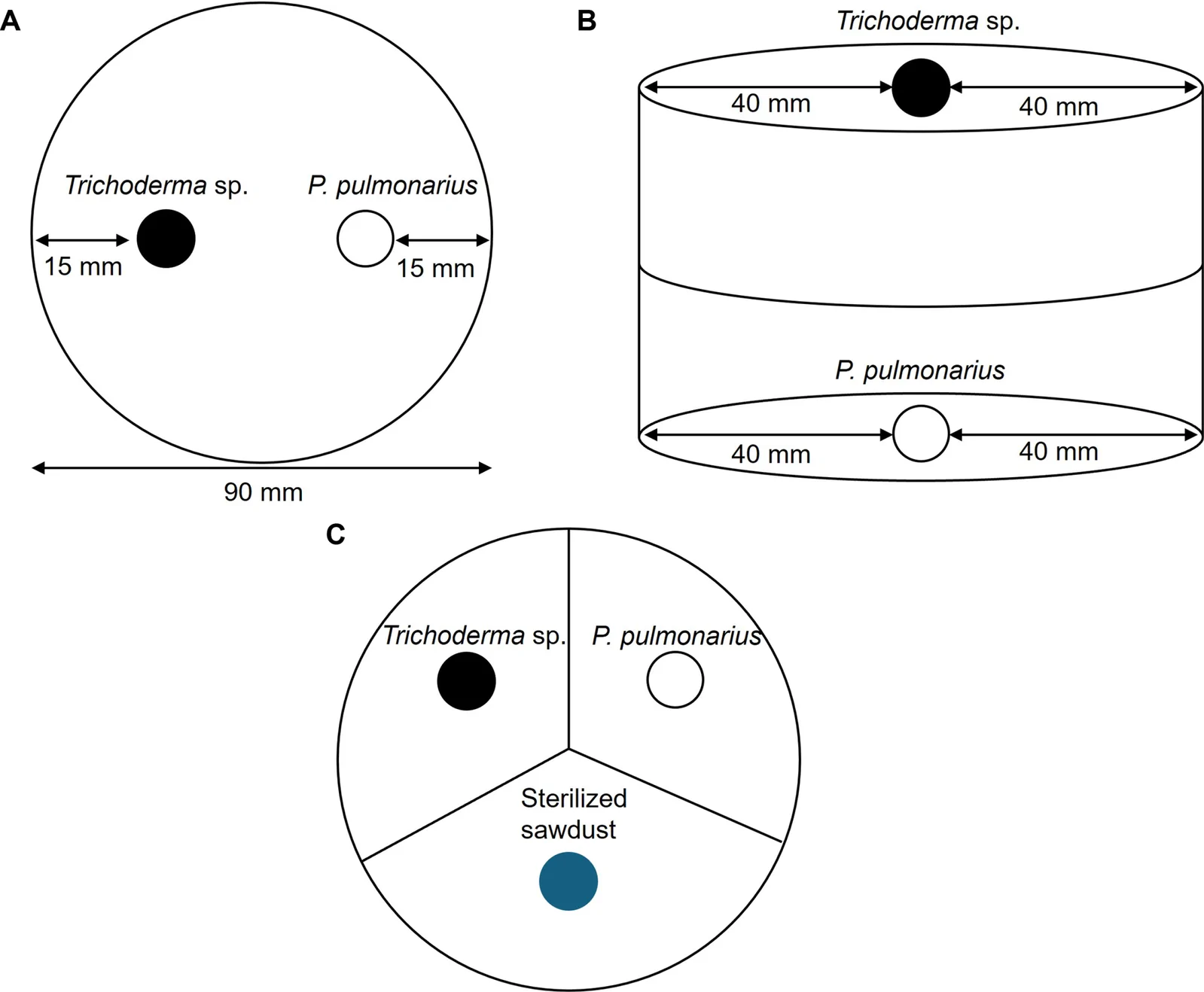
Overview of the experimental setup of (A) direct plate assay, (B) inverted plate assay, and (C) tripartite plate assay between two mycelia plugs.

**Fig. 2:**
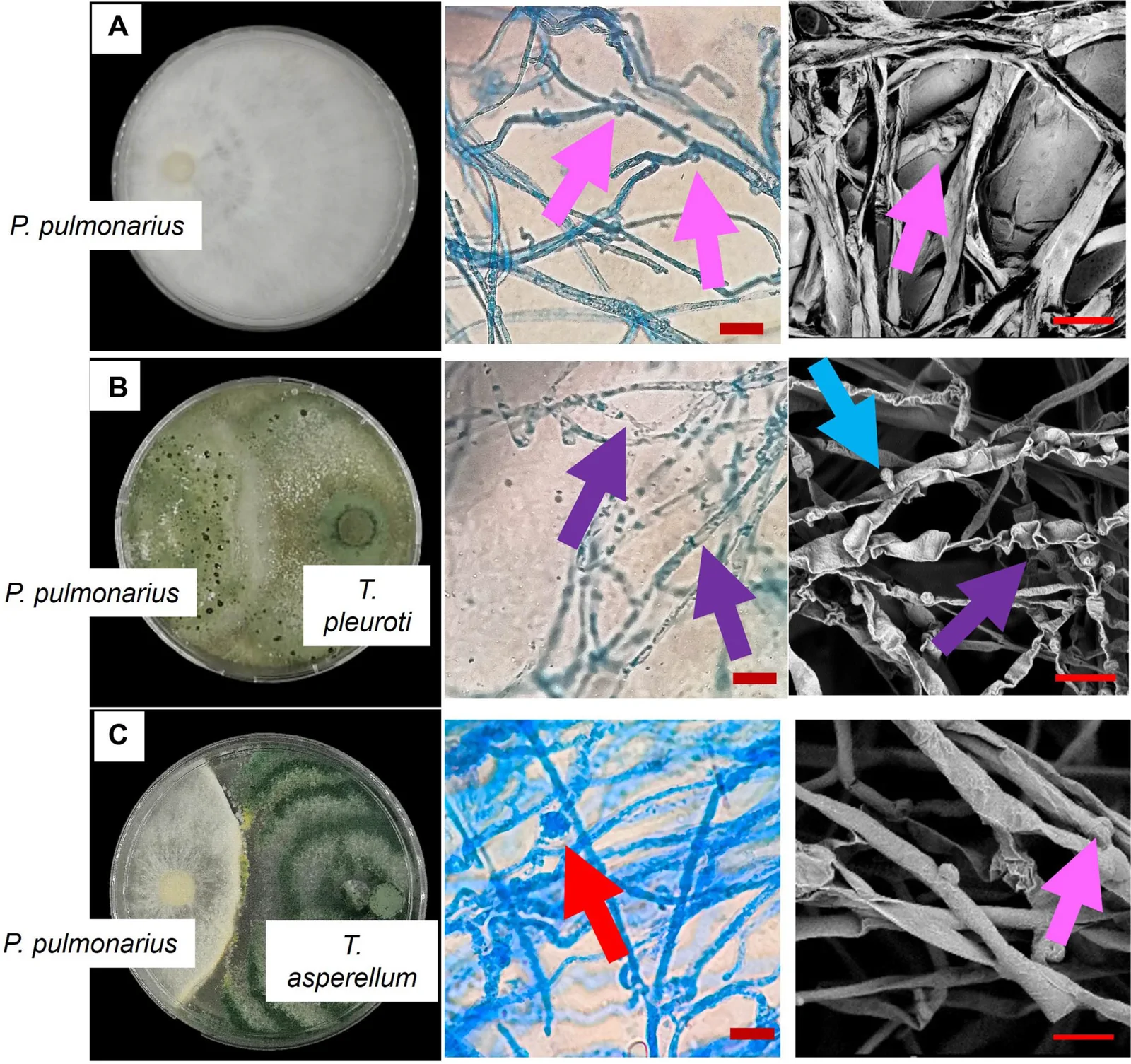
Direct plate assay of inhibition effect of *Trichoderma* spp. against *Pleurotus pulmonarius*. *Pleurotus* was inoculated on the left side, while *Trichoderma* was inoculated on the right side of the Petri dish. The left panel showed the macroscopic morphology of the inhibitory effect, the middle panel showed the microscopic morphology at 400x magnification, and the right panel showed scanning electron microscopy images at 5000x magnification (Bar scale = 10 μm). (**A**) Control plate for *P. pulmonarius*; (**B**) Inhibitory effect of *T. pleuroti* against *P. pulmonarius*; (**C**) Inhibitory effect of *T. asperellum* against *P. pulmonarius*. Pink arrows indicate generative hyphae of *Pleurotus* with clamp connections; purple arrows indicate lysis of hyphae; blue arrow indicates the formation of specialized structures of *Trichoderma* on the hyphal surface of *Pleurotus* and red arrow indicates the coiling of hyphae

**Fig. 3:**
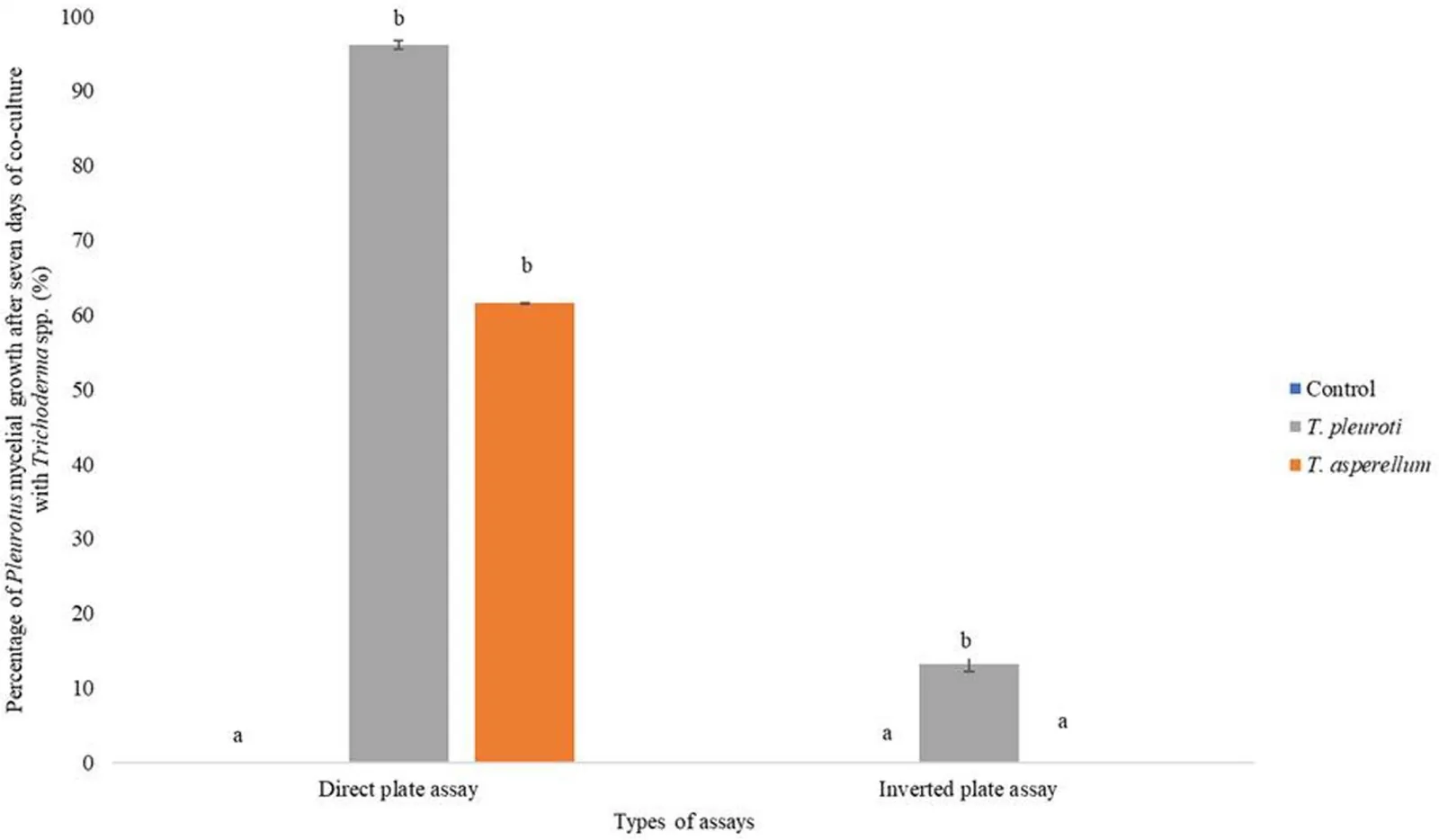
Percentage (%) of *P. pulmonarius* mycelial growth inhibition after seven days of co-cultured with *T. pleuroti* and *T. asperellum*. Different letters indicate significant differences based on Duncan’s Multiple Range Test (DMRT) (*p*<0.05).

**Fig. 4:**
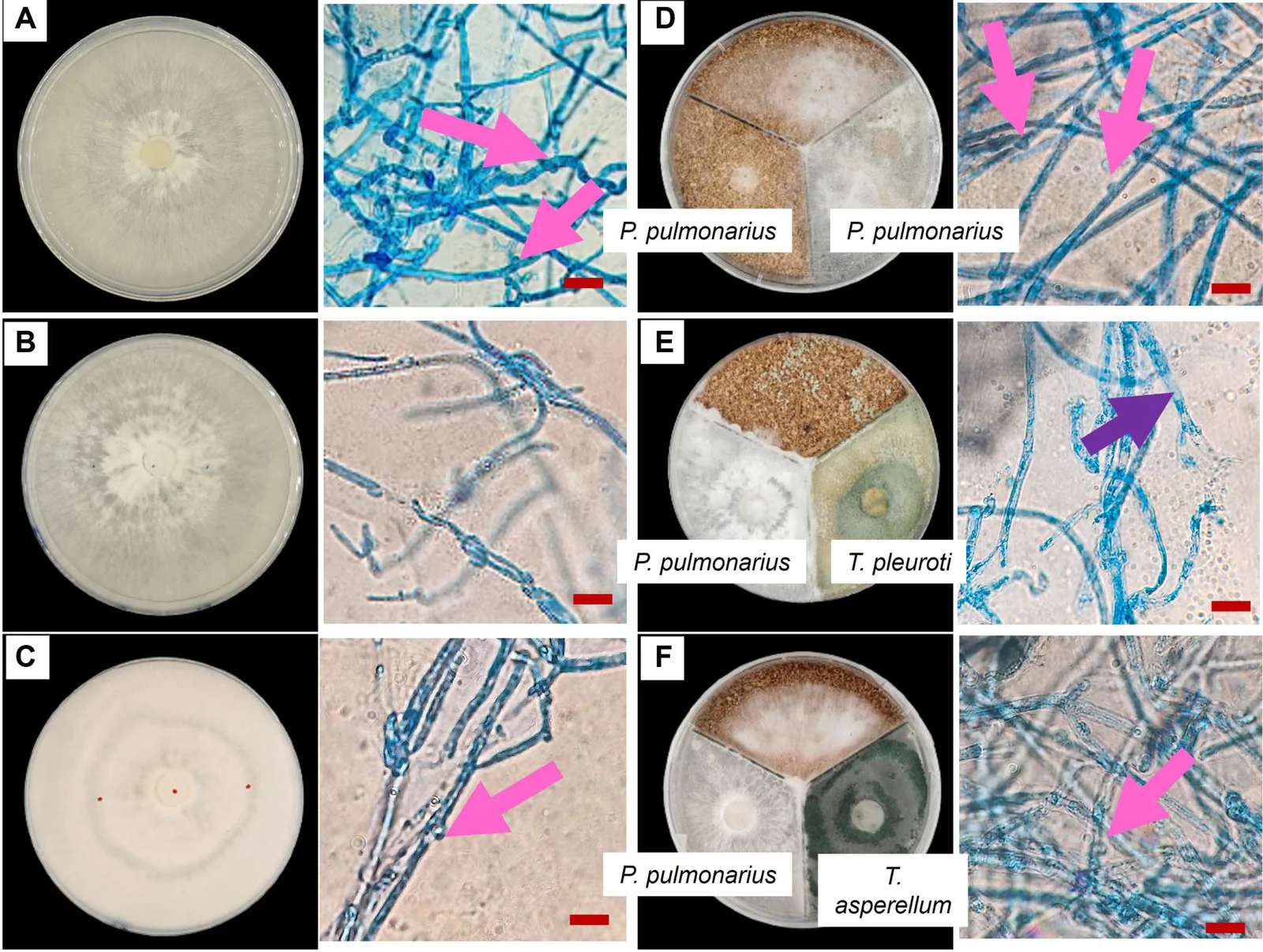
Inhibitory effect of *Trichoderma* spp. against *Pleurotus pulmonarius* in (A-C) inverted plate assay and (D-F) tripartite plate assay. The left panel showed the macroscopic morphology of the inhibitory effect, and right panel showed the microscopic morphology at 400x magnification (Bar scale = 10 μm). (**A, D**) Control plate for *P. pulmonarius*; (**B, E**) Inhibitory effect of *T. pleuroti* against *P. pulmonarius*; (**C, F**) Inhibitory effect of *T. asperellum* against *P. pulmonarius*. Pink arrows indicate generative hyphae of *Pleurotus* with clamp connections and purple arrows indicate lysis of *Pleurotus* hyphae

**Fig. 5:**
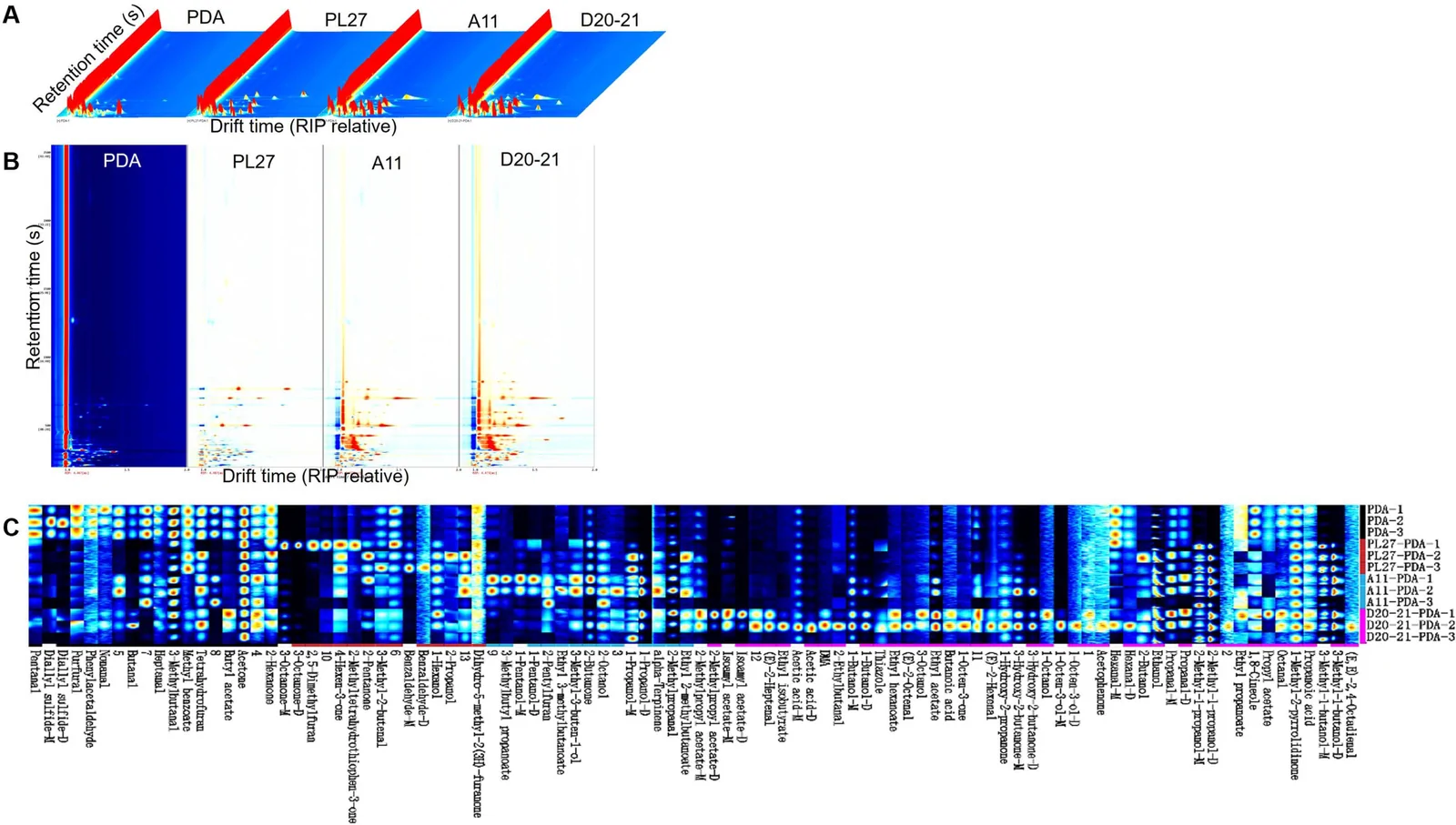
Variation in VOC signal abundance across *Pleurotus* and *Trichoderma* in PDA medium by HS-GC-IMS. (**A**) 3D-topography plot of collected signal; (**B**) 2D-topograph where the spectra of PL27, All and D20-21 were compared with the PDA (reference); and (**C**) Fingerprints of gallery plot. If the VOC in the target sample is same as the reference, the background after subtraction appears white. Red indicates that the concentration of the VOC is higher than the reference while blue indicates a lower concentration of the VOC than the reference. Each point on the reaction ion peak (RIP) represents a volatile organic compound. PDA: potato dextrose agar medium which was used to inoculate fungal mycelium; PL27: *Pleurotus pulmonarius* mycelium; A11: *T. asperellum*; D20-21: *T. pleuroti* .

**Fig. 6:**
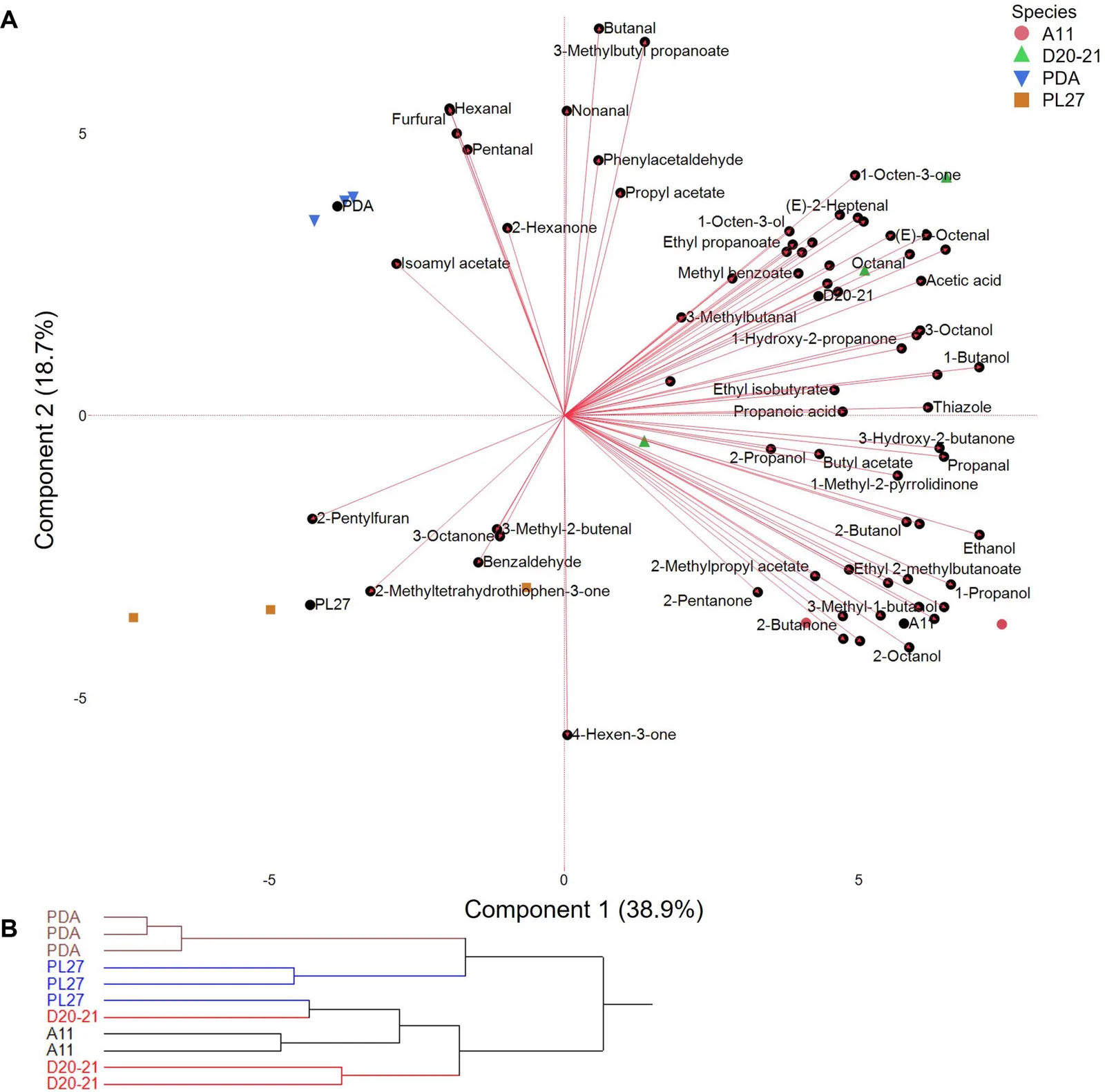
(A) PCA biplot of VOCs and (B) one-way cluster dendrogram of VOCs in *P. pulmonarius*, *T. pleuroti*, and *T. asperellum*. PDA: potato dextrose agar medium which was used to inoculate fungal mycelium; PL27: *Pleurotus pulmonarius* mycelium; A11: *T. asperellum*; D20-21: *T. pleuroti* (Note: One replicate of *T. asperellum* outlier was excluded from PCA and HCA analysis).

**Fig. 7:**
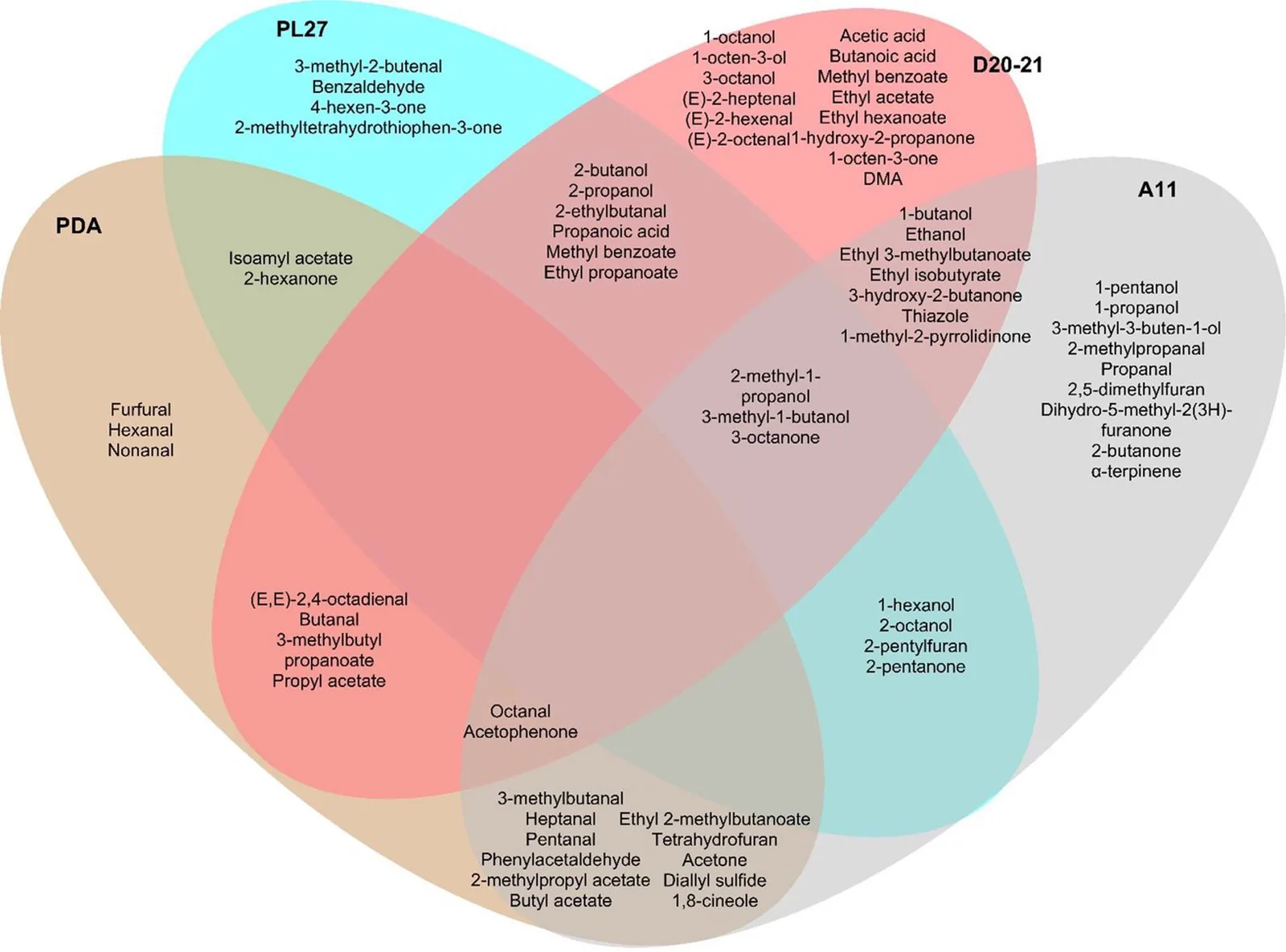
Venn diagram showed the PLS-DA coefficients of VOCs detected in each sample type: PDA medium (PDA), *P. pulmonarius* (PL27), *T. pleuroti* (D20-21), and *T. asperellum* (A11).

**Table 1:** Inhibitory effect, frequency of inhibition, and percentage of growth inhibition calculated on *Pleurotus pulmonarius* by *Trichoderma* spp. in direct plate assay.

*Trichoderma* spp.	Frequency	*Pleurotus pulmonarius*			Competition	Percentage of total inhibitory index (%)
		IIE^a^	GIE^b^	II^c^		
*T. pleuroti*	3	+4	+12	BA2^d^	Replacement of mushrooms by pathogenic fungi	78
*T. asperellum*	3	0	0	A^e^	Deadlock	22
Total	6	-	-	13.5	-	100

^a^IIE – individual inhibitory effect, ^b^GIE – general inhibitory effect, ^c^II – inhibitory index, ^d^BA2 – complete replacement after initial deadlock with contact, ^e^A – deadlock with mycelial contact

**Table 2:** Discriminatory VOCs associated with *T. pleuroti* and reported antifungal activity.

Chemical class	VOC	Literature target organism(s)	Reported antifungal effect (literature)	VIP score^a^	References	Reported *Trichoderma* VOCs producers
Alcohol	1-octanol	*Aspergillus flavus*	Damage the cell membrane	1.0	[46]	*T. harzianum* [41]
	1-octen-3-ol	*Agaricus bisporus*, *Penicillium chrysogenum*	Affect the balance of nutrients and metabolites	0.9	[40]	*T. harzianum*, *T. virens* [41]
	3-octanol	*A. flavus*, *Botrytis cinerea*, *Fusarium verticillioides*	Reduce cell viability	0.9	[47]	*T. longibrachiatum*, *T. virens* [42]
Aldehyde	(E)-2-heptenal	*A. flavus*	Disrupt plasma membrane integrity	1.0	[36]	N/A^b^
	(E)-2-hexenal	*A. flavus*, *B. cinerea*	Disrupt mitochondrial energy	0.8	[35]	N/A
	(E)-2-octenal	*Penicillium italicum*	Damage cell membrane and mitochondrial dysfunction	1.0	[37]	N/A
Carboxylic acid	Acetic acid	*Cercospora beticola*, *Colletotrichum lagenarum*, *Sclerotinia sclerotiorum*	Inhibit their growth	0.9	[14]	*T. harzianum*, *T. longibrachiatum* [42], *T. virens* [41]
	Butanoic acid	*C. beticola*, *C. lagenarum*, *S. sclerotiorum*	Inhibit their growth	1.0	[14]	*T. asperellum*, *T. harzianum*, *T. virens* [42]
	Propanoic acid	*Mycosphaerella fijiensis*, *S. sclerotiorum*, *Phytophthora cinnamomic*, *Pythium ultimum*	DNA damage in cells	1.0	[34]	*T. afroharzianum* [43]
Ester	Methyl benzoate	*Candida albicans*, *C. krusei*, and *C. parapsilosis*	Multi-target binding to enzymes	0.8	[39]	*T. longibrachiatum* [42]
	Ethyl acetate	*C. albicans*	Inhibit germ tube formation	1.0	[38]	*T. atroviride*, *T. harzianum*, *T. virens* [42]
Ketone	1-hydroxy-2-propanone	*Schizophyllum commune*	Inhibit the fungal growth	1.0	[48]	*T. koningiopsis* [44]

^a^VIP (Variable Importance in Projection) from the PLS-DA model; higher scores indicate stronger contribution to separating *T. pleuroti* from the comparison set

^b^N/A (not reported in other *Trichoderma* species)
