# Supplementary material for: Dual Evidence of Trichoderma pleuroti Antagonism against Pleurotus pulmonarius: Direct Inhibition and Volatile-Mediated Bioassays with Volatilome Fingerprinting
Source: J Microbiol Biotechnol. 2026 Sep 21;36:e2605010. doi: 10.4014/jmb.2605.05010 (PMC13669513; doi:10.4014/jmb.2605.05010)
Supplement: Supplementary file 1 [file jmb-36-e2605010-supple.pdf]

J. Microbiol. Biotechnol. 2026. 36: e2605010
https://doi.org/10.4014/jmb.2605.05010

Supplementary Figure and Tables

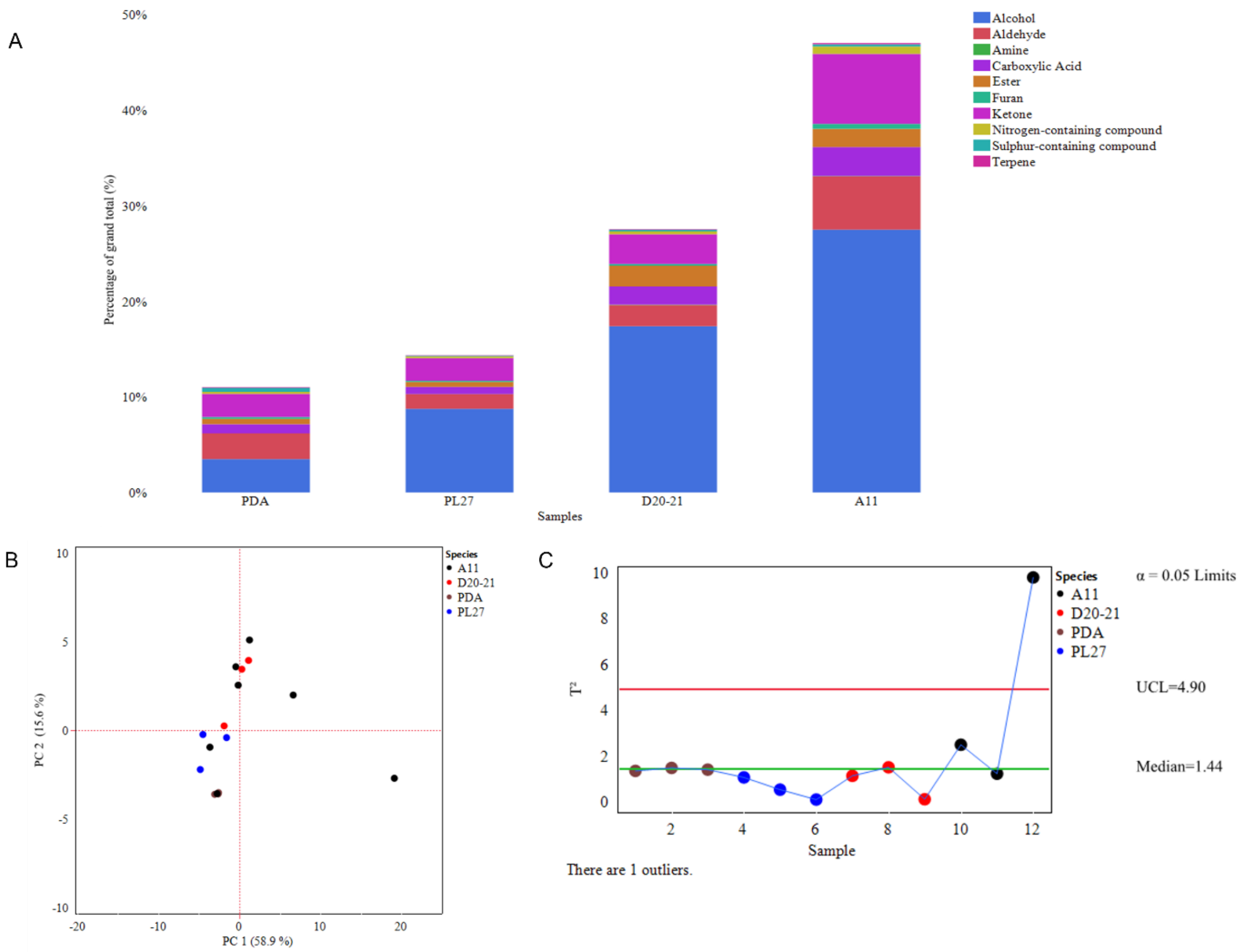

Fig. S1. Multivariate analysis of VOCs in PDA medium (PDA), *P. pulmonarius* (PL27), *T. pleuroti* (D20-21), and *T. asperellum* (A11). (A) Percentage (%) of grand total of concentration of volatile compound categories. **(B)** Scores plot for PCA model. **(C)** Outlier analysis at α=0.05

Table S1. Scale for evaluating the situation in dual fungi culture (Mańka, 1994) and Inhibitory index (II) of *Pleurotus* by *Trichoderma* (Badalyan *et al.,* 2002)

	Situation	Score
	**1. Surrounding of colony**	
A)	Both fungal colonies, i.e. of *Trichoderma* and of *Pleurotus* meeting along a straight line	0
B)	*Trichoderma* meeting *Pleurotus* along a slightly curved line surrounding less than 1/3 of it	+1
C)	*Trichoderma* meeting *Pleurotus* along a curved line surrounding at least 1/3, but less than 1/2 of it	+2
D)	*Trichoderma* meeting *Pleurotus* along a curved line surrounding at least ½ but less than 2/3 of it	+3
E)	*Trichoderma* meeting *Pleurotus* along a curved line surrounding 2/3 or more of it	+4
	**2. Inhibition zone**	
A)	Each millimeter of inhibition zone between partner colonies in favor of *Trichoderma*	+1
	**3. Reduction of colony size**	
A)	*Pleurotus* at least 1/3 but less than ½ smaller than its separately-grown control colony	+1
B)	*Pleurotus* at least 1/2 but less than 2/3 smaller than its separately-grown control colony	+2
C)	*Pleurotus* at least 2/3 smaller than its separately-grown control colony	+3
D)	*Pleurotus* completely undeveloped	+4
**Inhibitory index (II)**		
**A**	deadlock with mycelial contact	1
**B**	overgrowth without initial deadlock	2
**BA1**	partial replacement after initial deadlock with contact	2.5
**BA2**	complete replacement after initial deadlock with contact	3.5

Table S2. Volatile organic compounds detected in *P. pulmonarius* (PL27), *T. pleuroti* (D20-21) and *T. asperellum* (A11) inoculated on PDA

Category	Compounds	Rt[a] (s)	RI[b]	Dt[c]	Concentration (mg/kg)		
					PL27	D20-21	A11
Alcohol	1-butanol	595.89	1165.70	1.38	1.35 ± 0.23[a]	7.26 ± 0.82[b]	6.13 ± 0.23[b]
	1-hexanol	957.69	1375.70	1.34	0.30 ± 0.09[a]	0.43 ± 0.08[a]	0.50 ± 0.09[a]
	1-octanol	1779.61	1662.50	1.48	0.52 ± 0.14[a]	0.96 ± 0.24[a]	0.72 ± 0.07[a]
	1-octen-3-ol	1230.73	1491.80	1.60	0.42 ± 0.13[a]	5.46 ± 3.50[a]	0.69 ± 0.10[a]
	1-pentanol	763.40	1269.30	1.51	0.51 ± 0.09[a]	0.67 ± 0.05[a]	3.36 ± 0.88[b]
	1-propanol	423.52	1060.20	1.25	6.32 ± 2.62[a]	16.93 ± 3.33[ab]	27.61 ± 4.24[b]
	2-butanol	407.11	1046.50	1.34	0.06 ± 0.02[a]	0.11 ± 0.01[a]	0.10 ± 0.01[a]
	2-methyl-1-propanol	498.32	1113.50	1.37	23.28 ± 7.40[a]	38.58 ± 0.05[ab]	53.79 ± 7.59[b]
	2-octanol	1109.77	1444.00	1.46	0.41 ± 0.08[a]	0.50 ± 0.08[ab]	0.80 ± 0.03[b]
	2-propanol	312.66	941.80	1.23	0.87 ± 0.39[a]	1.06 ± 0.29[a]	0.89 ± 0.18[a]
	3-methyl-1-butanol	698.35	1225.60	1.49	17.40 ± 6.09[a]	30.74 ± 0.92[ab]	42.14 ± 6.67[b]
	3-methyl-3-buten-1-ol	760.69	1267.60	1.17	0.20 ± 0.06[a]	0.23 ± 0.09[a]	0.68 ± 0.09[b]
	3-octanol	1100.26	1440.00	1.40	0.14 ± 0.03[a]	0.40 ± 0.09[ab]	0.26 ± 0.05[b]
	Ethanol	318.13	949.80	1.13	58.65 ± 21.06[a]	133.22 ± 7.29[ab]	167.80 ± 22.35[b]
Aldehyde	(E)-2-heptenal	880.79	1337.00	1.26	0.07 ± 0.01[a]	0.86 ± 0.03[b]	0.15 ± 0.00[c]
	(E)-2-hexenal	714.61	1236.90	1.18	0.19 ± 0.05[a]	0.48 ± 0.11[ab]	0.34 ± 0.06[b]
	(E)-2-octenal	1109.77	1444.00	1.34	0.19 ± 0.03[a]	0.62 ± 0.16[ab]	0.31 ± 0.03[b]
	(E,E)-2,4-octadienal	1817.64	1672.30	1.28	0.42 ± 0.09[a]	0.63 ± 0.15[a]	0.66 ± 0.11[a]
	2-ethylbutanal	377.20	1020.10	1.51	0.04 ± 0.01[a]	0.17 ± 0.10[a]	0.04 ± 0.01[a]
	2-methylpropanal	246.30	832.60	1.28	1.40 ± 0.56[a]	3.78 ± 1.44[ab]	5.02 ± 0.25[b]
	3-methyl-2-butenal	689.68	1219.50	1.09	0.28 ± 0.13[a]	0.09 ± 0.01[a]	0.21 ± 0.01[a]
	3-methylbutanal	308.29	935.40	1.40	7.54 ± 2.57[a]	11.56 ± 4.15[ab]	21.16 ± 1.64[b]
	Benzaldehyde	1416.75	1557.00	1.47	3.79 ± 1.87[a]	0.83 ± 0.01[a]	1.02 ± 0.12[a]
	Butanal	282.03	894.60	1.28	0.08 ± 0.02[a]	2.45 ± 0.95[a]	0.58 ± 0.02[b]
	Furfural	1251.24	1499.50	1.09	0.10 ± 0.03[a]	0.15 ± 0.02[a]	0.15 ± 0.02[a]
	Heptanal	663.11	1200.20	1.36	0.09 ± 0.02[a]	0.16 ± 0.00[a]	0.24 ± 0.00[b]

Table S2. (continued)

Aldehyde	Hexanal	488.56	1107.70	1.56	0.52 ± 0.10[a]	0.77 ± 0.31[a]	0.43 ± 0.10[a]
	Nonanal	1032.00	1410.30	1.49	0.13 ± 0.04[a]	0.20 ± 0.01[a]	0.19 ± 0.02[a]
	Octanal	822.99	1305.60	1.42	0.07 ± 0.02[a]	0.18 ± 0.02[b]	0.17 ± 0.00[b]
	Pentanal	362.62	1006.40	1.42	0.04 ± 0.01[a]	0.07 ± 0.00[a]	0.08 ± 0.04[a]
	Phenylacetaldehyde	2245.97	1770.20	1.26	0.59 ± 0.14[a]	0.89 ± 0.04[ab]	1.11 ± 0.04[b]
	Propanal	241.55	823.70	1.15	3.80 ± 0.85[a]	8.08 ± 0.90[ab]	9.71 ± 2.16[b]
Amine	Dimethylacetamide (DMA)	1050.14	1418.40	1.35	0.15 ± 0.04[a]	0.70 ± 0.15[a]	0.24 ± 0.02[b]
Carboxylic Acid	Acetic acid	1269.02	1506.00	1.16	8.78 ± 2.06[a]	23.91 ± 7.04[a]	16.54 ± 1.48[a]
	Butanoic acid	1909.71	1695.20	1.17	0.25 ± 0.05[a]	0.64 ± 0.00[a]	0.43 ± 0.04[b]
	Propanoic acid	1689.54	1638.50	1.12	0.38 ± 0.11[a]	0.47 ± 0.06[a]	0.53 ± 0.08[a]
Ester	Methyl benzoate	2159.90	1752.10	1.20	0.44 ± 0.15[a]	4.26 ± 1.85[ab]	0.84 ± 0.11[b]
	2-methylpropyl acetate	394.71	1035.80	1.61	0.05 ± 0.01[a]	0.07 ± 0.00[a]	0.45 ± 0.15[b]
	3-methylbutyl propanoate	648.48	1190.40	1.83	0.02 ± 0[a]	0.16 ± 0.03[a]	0.04 ± 0.01[b]
	Butyl acetate	469.10	1095.70	1.24	1.13 ± 0.24[a]	1.27 ± 0.08[a]	1.65 ± 0.24[a]
	Ethyl 2-methylbutanoate	439.92	1073.40	1.26	0.04 ± 0.01[a]	0.06 ± 0.01[a]	0.14 ± 0.02[b]
	Ethyl 3-methylbutanoate	457.06	1086.60	1.27	2.40 ± 0.10[a]	23.68 ± 0.29[b]	16.36 ± 0.83[c]
	Ethyl acetate	288.96	905.70	1.34	0.04 ± 0.01[a]	0.19 ± 0.03[a]	0.07 ± 0.00[b]
	Ethyl hexanoate	739.01	1253.40	1.33	0.04 ± 0.01[a]	0.38 ± 0.23[a]	0.08 ± 0.01[a]
	Ethyl isobutyrate	346.57	989.00	1.56	0.01 ± 0[a]	0.03 ± 0.01[a]	0.03 ± 0.01[a]
	Ethyl propanoate	341.83	982.70	1.45	0.52 ± 0.11[a]	6.68 ± 2.36[a]	0.67 ± 0.15[b]
	Isoamyl acetate	551.44	1143.10	1.75	2.04 ± 0.71[a]	1.45 ± 0.12[a]	1.26 ± 0.21[a]
	Propyl acetate	356.78	1000.80	1.48	0.03 ± 0.01[a]	0.09 ± 0.07[a]	0.04 ± 0.00[a]
Furan	2-pentylfuran	733.04	1249.40	1.25	0.16 ± 0.12[a]	0.02 ± 0.01[a]	0.03 ± 0.00[a]
	2,5-dimethylfuran	322.51	956.00	1.37	0.17 ± 0.06[a]	0.34 ± 0.00[a]	0.71 ± 0.08[b]
	Dihydro-5-methyl-2(3H)-furanone	2105.86	1740.40	1.13	0.04 ± 0.01[a]	0.06 ± 0.00[a]	0.08 ± 0.01[a]
	Tetrahydrofuran	269.63	874.00	1.23	1.14 ± 0.54[a]	2.18 ± 0.58[a]	2.79 ± 0.16[a]

Table S2. (continued)

Ketone	1-hydroxy-2-propanone	845.98	1318.30	1.23	0.10 ± 0.03^{a}	0.23 ± 0.02^{a}	0.19 ± 0.03^{a}
	1-octen-3-one	843.35	1316.90	1.28	0.05 ± 0.01^{a}	0.15 ± 0.04^{ab}	0.08 ± 0.00^{b}
	2-butanone	299.17	921.60	1.24	3.54 ± 0.95^{a}	3.57 ± 1.24^{a}	11.91 ± 3.19^{b}
	2-hexanone	483.32	1104.60	1.19	0.15 ± 0.02^{a}	0.15 ± 0.01^{a}	0.17 ± 0.05^{a}
	2-pentanone	361.89	1005.70	1.37	0.79 ± 0.18^{a}	0.69 ± 0.18^{a}	1.13 ± 0.66^{a}
	3-hydroxy-2-butanone	821.02	1304.50	1.33	1.34 ± 0.60^{a}	8.65 ± 1.54^{b}	8.64 ± 2.99^{b}
	3-octanone	767.80	1272.20	1.72	5.14 ± 4.43^{a}	4.00 ± 0.72^{a}	2.61 ± 0.22^{a}
	4-hexen-3-one	667.45	1203.40	1.11	0.21 ± 0.06^{a}	0.05 ± 0.01^{a}	0.16 ± 0.01^{a}
	Acetone	251.04	841.30	1.11	18.04 ± 4.31^{a}	17.36 ± 1.50^{a}	25.15 ± 4.74^{a}
	Acetophenone	2288.00	1778.80	1.17	0.33 ± 0.07^{a}	0.54 ± 0.10^{a}	0.56 ± 0.08^{a}
Nitrogen-containing compound	1-methyl-2-pyrrolidinone	2428.11	1806.30	1.10	2.46 ± 0.63^{a}	3.25 ± 0.07^{a}	4.25 ± 0.67^{a}
Sulphur-containing compound	Diallyl sulfide	561.74	1148.50	1.32	0.20 ± 0.02^{a}	1.66 ± 0.44^{ab}	1.01 ± 0.08^{b}
	2-methyltetrahydrothiophen-3-one	1539.42	1595.40	1.21	0.70 ± 0.21^{a}	0.34 ± 0.06^{a}	0.49 ± 0.09^{a}
	Thiazole	774.37	1276.30	1.24	0.07 ± 0.03^{a}	0.39 ± 0.10^{ab}	0.29 ± 0.07^{b}
Terpene	1,8-cineole	693.47	1222.20	1.30	0.50 ± 0.15^{a}	0.89 ± 0.05^{a}	1.04 ± 0.11^{a}
	α-terpinene	666.91	1203.00	1.22	0.06 ± 0.02^{a}	0.07 ± 0.01^{a}	0.14 ± 0.01^{b}

[a] Rt: Retention time (seconds); [b] RI: Retention index calculated using 2-butanone, 2-pentanone, 2-hexanone, 2-heptanone, 2-octanone, and 2-nonanone as external standard; [c] Dt: Drift time; Data concentration presented as “mean ± standard error”; Different letters indicate significant differences based on Duncan’s Test (DMRT) (p<0.05).
